# Bipolar Disorder in Children

**DOI:** 10.1155/2014/928685

**Published:** 2014-02-24

**Authors:** Kimberly Renk, Rachel White, Brea-Anne Lauer, Meagan McSwiggan, Jayme Puff, Amanda Lowell

**Affiliations:** University of Central Florida, P.O. Box 161390, Orlando, FL 32816, USA

## Abstract

Although bipolar disorder historically was thought to only occur rarely in children and adolescents, there has been a significant increase in children and adolescents who are receiving this diagnosis more recently (Carlson, 2005). Nonetheless, the applicability of the current bipolar disorder diagnostic criteria for children, particularly preschool children, remains unclear, even though much work has been focused on this area. As a result, more work needs to be done to further the understanding of bipolar symptoms in children. It is hoped that this paper can assist psychologists and other health service providers in gleaning a snapshot of the literature in this area so that they can gain an understanding of the diagnostic criteria and other behaviors that may be relevant and be informed about potential approaches for assessment and treatment with children who meet bipolar disorder criteria. First, the history of bipolar symptoms and current diagnostic criteria will be discussed. Next, assessment strategies that may prove helpful for identifying bipolar disorder will be discussed. Then, treatments that may have relevance to children and their families will be discussed. Finally, conclusions regarding work with children who may have a bipolar disorder diagnosis will be offered.

## 1. Bipolar Disorder in Children

Although bipolar disorder historically was thought to only occur rarely in children and adolescents, there has been a significant increase in children and adolescents who are receiving this diagnosis currently [1, 2]. In fact, a recent meta-analysis suggested that the overall occurrence of bipolar disorder in children and adolescents was 1.8% [3]. Nonetheless, the applicability of the current bipolar disorder diagnostic criteria for children, particularly preschool children, remains unclear, even though much work has been focused on this area. As a result, more work needs to be done to further the understanding of bipolar symptoms in children. It is hoped that this paper can assist psychologists and other health service providers in gleaning a snapshot of this diagnosis so that they can gain an understanding of the diagnostic criteria and other behaviors that may be relevant and be informed about potential approaches for assessment and treatment with these children. First, the history of bipolar symptoms and current diagnostic criteria will be discussed. Next, assessment strategies that may prove helpful for identifying bipolar disorder will be discussed. Then, treatments that may have relevance to children and their families will be discussed. Finally, conclusions regarding work with children who have a bipolar disorder diagnosis will be offered.

## 2. History of Bipolar Disorder

Although the identification of bipolar symptoms (i.e., depression and mania) may be relatively recent in children, the identification of these symptoms is certainly not new. In fact, depression and mania appear to be the world's first documented mental illnesses [4], tracing back to ancient Greece [5]. For example, Hippocrates considered melancholia (i.e., depression) and mania to be among the earliest diagnosable disorders. In the first century A.D., the Greek physician Aretaeus of Cappadocia combined these two groups of symptoms into bipolar disorder by stating that mania was a worsened state of melancholia (rather than suggesting that mania and melancholia were distinct [4]). In Aretaeus' texts *On the Aetiology and Symptomatology of Chronic Diseases* and *The Treatment of Chronic Diseases*, sufferers of melancholia were depicted as quiet or dysphoric and sad or apathetic, and sufferers of mania were depicted as cheerful [4]. Other references did not draw a connection between melancholia and mania until the seventeenth century; however, for example, Théophile Bonet began using the term *melancholicus mania* in 1679 [6] and Willis described melancholia and mania as *distempers of raving* in his writing [7].

Following these initial references to bipolar symptoms, advancements in the classification of bipolar disorder generally did not occur until the 19th century. For example, in 1851, a new reference to bipolar disorder was made. At this time, French psychiatrist Jean-Pierre Falret conceptualized bipolar disorder as being cyclical in nature, referring to this phenomenon as the *folie circulaire* (i.e., circular madness). Falret described manic and melancholic episodes that were separated by symptom-free intervals [4, 5]. In 1854, French psychiatrist Jules Baillarger also described the cycling of manic and melancholic symptoms (i.e., *folie à double forme*), but without the presence of symptom-free intervals [4, 5]. Although Falret and Baillarger disagreed on the exact nature and cyclical process of bipolar disorder, they both agreed that the diagnosis and prognosis of this disorder was “desperate, terrible, and incurable” [8, p. 405]. This early thinking allowed for bipolar disorder to be accepted throughout Europe.

During the end of the 19th century and the beginning of the 20th century, Emil Kraepelin employed a unifying approach to the classification of mood disorders, resulting in bipolar disorder being subsumed within the category of *manic-depressive insanity (MDI)*. MDI included *circular insanity* and unipolar disorders (e.g., single-episode and recurring depression [9]). Unlike previous descriptions of bipolar symptoms, the diagnosis of MDI had a good prognosis [9]. Although Kraepelin suggested that bipolar disorder was a severe mental illness, he also suggested that individuals with this diagnosis experienced mild residual states after recovery from individual episodes and mild fluctuations between episodes. It is also noteworthy that Kraepelin referred to the possibility of manic depression occurring in children, although rarely [10].

Because Kraepelin's unification of mood disorders was so widespread, further research on the course of these disorders failed to differentiate between depression, mania, and bipolar disorder [11]. Nonetheless, there were many psychiatrists who opposed Kraepelin's idea of merging mood disorders into one category. In particular, German psychiatrists Wernicke [12], Kleist [13], and Leonhard [14] described subtle differences in various mood syndromes and described them as separate entities. Together, Kleist and Leonhard gathered data on family history and clinical course to support the distinction between *bipolar disorders* and *unipolar disorders* [9]. This information would not be taken seriously in psychiatry until 1966, the year referred to as the “rebirth” of bipolar disorders [15] due to the publication of two important works.

These works were Angst's [16] monograph and Perris and d'Elia's [17] article [15]. Both works suggested that genetics played a role in the etiology of endogenous depression, that bipolar disorder was represented equally in males and females, that manic-depressive illness was not homogenous (i.e., unipolar depression differed significantly from bipolar disorder), and that unipolar depression had a strong genetic relationship to bipolar disorder [15]. These works, along with research completed by Winokur and colleagues [18], confirmed the findings postulated by Kleist and his colleagues. As a whole, these works contributed to the increased interest in and knowledge of bipolar disorder in the 20th century.

With regard to theorizing about bipolar symptoms in children, psychoanalytic theorists, such as Adolf Meyer, Karl Abraham, and Melanie Klein, were some of the first to make references to manic-depressive symptoms in children (see [5], for a summary). In fact, child psychiatrists began to examine bipolar symptoms in their patients, looking for presentations that resembled those seen in adults. Case studies then began to suggest that bipolar symptoms in children were rare and that, if these symptoms did occur, they generally did not occur until late adolescence [5]. In contrast, some psychiatrists suggested that mania was being overlooked in children because of the overlap between manic symptoms and those of other disorders (e.g., attention-deficit/hyperactivity disorder [ADHD]) and because of the appearance of manic symptoms in children who were developing typically. Although group studies and other studies of bipolar symptoms in children were documented by a variety of researchers starting in the 1920s, Anthony and Scott [19] concluded in a systematic review of the literature that manic depression was rare in childhood.

Beginning in the 1970s, however, interest in understanding and identifying bipolar disorder in children experienced a resurgence (see [5]). For example, Weinberg and Brumback [20] created criteria to diagnose mania in children. These criteria, which included symptoms of euphoric or irritable mood, hyperactive behavior, and flight of ideas, served as the basis for the *DSM-III* bipolar disorder criteria. Since 1980, *DSM* criteria have specified that adult criteria can be used to diagnose mania in children, with modifications based on differences in age and developmental stage [21]. Consequently, by the early 1980s, there was an increasing acceptance that children could present with bipolar symptoms. For example, Carlson [22] suggested that children with mania were described as exhibiting hyperactivity, an absence of discrete episodes, more irritability and emotional lability (as opposed to euphoria), and a relative lack of paranoia or grandiosity. Additionally, children who were experiencing the depressive phase of the disorder were thought to not display specific depressive episodes. Instead, they were described as being agitated or irritable in the same way as children with unipolar depression. Further, children who were older than 9 years of age were described as presenting with a more classic pattern of symptoms that was marked by discrete episodes, euphoria and irritability, and grandiosity [22].

Nonetheless, bipolar disorder still remains controversial for children. For example, the World Health Organization United States/United Kingdom study of manic depression and schizophrenia [23] showed that these two English-speaking countries made vastly different diagnoses for the same constellation of symptoms, prompting an increased interest in operationally defining criteria for these diagnoses. More recent research suggested that such findings still occur consistently, as research using case vignettes suggested that mental health professionals in the United States still are more likely to assign a bipolar disorder diagnosis than those in the United Kingdom [24].

Consequently, structured and semistructured interviews were developed, and researchers began to depend more on standardized criteria for diagnoses, using symptoms such as those in the *Research Diagnostic Criteria* [25] and the *Diagnostic and Statistical Manual of Mental Disorders* [26–30]. With the increased importance of defining criteria and the decreased specificity of the definition of *episode* in the *DSM-III-R*, the diagnosis of bipolar disorder became symptom driven in 1987. In contrast, the initial appearance of bipolar disorder in the *International Classification of Diseases (ICD)* defined the disorder in broader terms and focused on the whole picture of episodes or the course of the illness [31]. Thus, more clarity is needed.

## 3. Bipolar Disorder Symptoms in the *DSM-IV-TR*

Until recently, the criteria that were described in the *DSM-IV* [28] and *DSM-IV-TR* [29] were used to diagnose bipolar disorder in the United States, with bipolar disorder being listed as a mood disorder (similar to the rubric proposed by Kraepelin [10]). In fact, there were several different diagnoses in the *DSM-IV-TR* bipolar diagnostic grouping, each made in conjunction with the presence of manic, mixed, or hypomanic episodes. A manic episode was identified when individuals experienced an episode of elevated, expansive, or irritable mood that lasted for at least one week (the duration could be less than one week if hospitalization was necessary [29]). This episode was accompanied by three or more symptoms (from seven provided symptoms) when the individual's mood was elevated or expansive and by four or more symptoms when the individual's mood was irritable. These symptoms could include grandiosity, a decreased need for sleep, pressured speech, flight of ideas or racing thoughts, distractibility, increased goal directed activity or psychomotor agitation, and excessive participation in pleasurable but risky activities. These symptoms had to cause severe impairment in functioning and could not be due to a substance or a general medical condition [29].

In contrast, for a mixed episode, individuals had to experience symptoms consistent with both a manic episode (as described above) and a depressive episode (with either depressed or sad mood or a lack of interest or pleasure in things that were usually enjoyed as well as changes in eating and sleep habits, changes in motor activity, feelings of worthlessness and guilt, lack of concentration, and suicidal ideation or behavior being experienced) for a duration of at least one week. These symptoms had to cause severe impairment in functioning and could not be due to a substance or a general medical condition [29]. Finally, for a hypomanic episode, individuals had to experience at least four days of elevated, expansive, or irritable mood accompanied by the same manic symptoms noted previously. These symptoms could not be due to a substance or a general medical condition [29].

Then, based on the mood episodes that were identified, diagnoses of bipolar I disorder, bipolar II disorder, and cyclothymia as well as bipolar disorder not otherwise specified could be made [29]. To receive a diagnosis of bipolar I disorder, individuals had to experience one or more manic or mixed episodes [29]. To receive a diagnosis of bipolar II disorder, individuals had to experience one or more depressive episodes and one or more hypomanic episodes (but no manic or mixed episodes). In the case of both bipolar I and bipolar II diagnoses, the episodes that were present could not be better accounted for by other diagnoses, and the symptoms had to cause clinically significant distress or impairment [29]. Further, to receive a diagnosis of cyclothymia, individuals had to experience numerous periods of hypomanic symptoms and numerous periods of depressive symptoms, such that there were no symptom-free periods that occurred for longer than two months at a time. Time frames varied for cyclothymia, with adults needing to exhibit this pattern for at least two years and children needing to exhibit this pattern for at least one year [29]. Finally, the category of bipolar disorder not otherwise specified was used when bipolar symptoms were experienced but did not fall clearly within any of the other categories listed here [29].

## 4. Bipolar Disorder Symptoms in the *DSM-5*

With the *DSM-5'*s release in May 2013, it now can be noted that the bipolar disorder diagnostic criteria look similar to those listed in the *DSM-IV-TR* [30]. In the *DSM-5*, depressive, manic, and hypomanic episodes are identified using similar criteria to those described above. In contrast, symptoms of mixed episodes now are replaced with a mixed features specifier (a specifier that can be used with any of the mood episodes [32]). This specifier is used when individuals experience full criteria for a manic episode and three symptoms of a depressive episode or when individuals experience full criteria for a depressive episode and three symptoms of a manic episode. This adaptation (i.e., the mixed features specifier) may allow for easier diagnosis in children.

With regard to the diagnoses themselves, the *DSM-5* again includes bipolar I disorder, bipolar II disorder, and cyclothymia. As in the *DSM-IV-TR*, individuals have to experience one or more manic episodes to receive a bipolar I disorder diagnosis, and individuals have to experience one or more depressive episodes and one or more hypomanic episodes (but not any manic episodes) to receive a bipolar II disorder diagnosis [30]. In addition to these traditional diagnoses, there are listings for substance/medication-induced bipolar and related disorder, bipolar and related disorder due to another medical condition, other specified bipolar and related disorder, and unspecified bipolar and related disorder [30], allowing for more latitude in making an appropriate diagnosis.

## 5. Other Bipolar-Related Symptoms That May Be of Relevance for Children

At this point, it should be clear that researchers have identified the experience of bipolar disorder in children and that *DSM *criteria are being used to make bipolar disorder diagnoses in children. Nonetheless, bipolar disorder still is considered to be rare in children by many (e.g., [33]), with some researchers reporting that many children are overlooked in the diagnosis of bipolar disorder due to a lack of clarity in the diagnostic criteria and concerns about the validity of this diagnosis in children [34, 35]. For example, although children in one sample of 36 consecutively hospitalized preschool children who were 3 to 5 years of age exhibited an irritable mood, evidenced a strong family history of affective illness, and had exhibited previous symptoms of ADHD, only 17% were diagnosed with bipolar disorder [36]. Further, given that some developmental differences in the manifestation of adult-onset versus childhood-onset bipolar disorder were identified (e.g., with children showing more mixed symptoms [37, 38]), many children may be assigned an incorrect diagnosis. Such difficulties in diagnosis may be particularly problematic, as accurate diagnosis is critical for avoiding mislabeling and unnecessary medication exposure [39]. In fact, a diagnosis of bipolar disorder not otherwise specified often may serve as a default, particularly when children may not exhibit a full constellation of symptoms or meet the prescribed symptom duration criterion [38, 40].

Nonetheless, the course of bipolar disorder symptoms in children hopefully will become clearer as more researchers begin to examine this issue. For example, Birmaher and colleagues [41] examined the four-year longitudinal course of bipolar symptoms in children and adolescents who ranged in age from 7 to 17 years and who had some constellation of bipolar symptoms. In this study, bipolar disorder not otherwise specified was diagnosed if a child exhibited clinically relevant bipolar symptoms that did not meet criteria for bipolar I disorder or bipolar II disorder [42]. Based on their findings, the majority of children who had been diagnosed with a bipolar spectrum disorder (81.5%) experienced a complete recovery from their symptoms by 2.5 years following their index episode, although 62.5% of these children experienced a recurrence of syndromal symptoms within an additional 1.5 year time period. The majority of these children with recurring symptoms experienced depressive symptoms or mixed presentations, with a much lower frequency of manic symptoms. A number of children moved from bipolar disorder not otherwise specified to bipolar I disorder or bipolar II disorder (38%) and from bipolar II disorder to bipolar I disorder (25% [41]). In addition to examining the course of bipolar disorder symptoms in this study, Birmaher and colleagues [41] identified several predictors of poorer outcomes, including an early onset of symptoms, a diagnosis of bipolar disorder not otherwise specified, a long duration of illness, a family history of mood disorders, and low socioeconomic status.

Given these considerations, it certainly may be challenging to identify bipolar disorder (as well as prodromes and early markers of the disorder) in children. Given the difficulties that children (particularly preschool children) face in verbalizing their emotions [42] and in recognizing and describing their own behaviors, researchers often may have to rely on the report of parents [33] or other informants and should look to evidence-based assessment recommendations [39]. Making distinctions between clinically significant symptoms and nonclinical behaviors still may be difficult, however. For example, distinguishing fantasies about special powers from clinically concerning levels of grandiosity and determining the cutoff for the frequency and/or intensity of euphoric states that should be of clinical concern may be more difficult in children (particularly preschool children [2, 33]). Nonetheless, research suggested that children as young as 3 years of age can manifest manic symptoms, with many parents claiming that these symptoms always had been present [33, 35, 43]. Given the relative certainty that bipolar *symptoms* do occur in children, further information beyond that provided in the *DSM* may be helpful.

### 5.1. Bipolar Phenotypes

Although formal diagnostic criteria are available, the National Institute of Mental Health (NIMH) organized a *Roundtable on Prepubertal Bipolar Disorder in Children* in 2000 [44] to provide further clarity regarding bipolar symptoms in children. By this time, there were at least a dozen different research groups investigating bipolar symptoms in children [45]. Participants in this roundtable agreed that the *Kiddie Schedule for Affective Disorders and Schizophrenia (K-SADS)* should be used to diagnose childhood-onset bipolar disorder, that children could present with *broad* or *narrow* phenotypes of bipolar disorder, and that bipolar disorder in children was highly comorbid with ADHD and other behavior disorders. A major issue that remained unresolved, however, was the assessment of mood variability and its convergence with bipolar I disorder, bipolar disorder not otherwise specified, and other disorders entirely [45].

Nonetheless, this roundtable prompted further research. One of the lines of thinking that came from this roundtable was that of Leibenluft and colleagues [46], who described a more uniform terminology for describing bipolar phenotypes, suggesting that childhood-onset bipolar disorder should be defined with several subtypes. These subtypes included the *narrow *subtype (for those who exhibited distinct mood episodes with hypomania or mania and elevated mood or grandiosity, with these episodes being more narrowly defined but less researched than those in the *DSM*), two *intermediate* subtypes (for those who exhibited clear episodes with hallmark *DSM *symptoms of hypomania or mania but who did not meet the duration criteria for these episodes and for those who exhibited irritable but not elevated mood), and the *broad *subtype (for those who exhibited chronic, nonepisodic symptoms of irritability and hyperarousal). Other researchers developed different rubrics. For example, Geller and Luby [47] suggested that childhood-onset bipolar disorder manifested with chronic, nonepisodic, rapid cycling, mixed manic states could cooccur with ADHD and conduct disorder. Further, they required euphoric mood and grandiosity to be present. Further, Wozniak and colleagues [48] emphasized that severe irritability was the predominant mood state and that grandiosity was not overrepresented in childhood-onset bipolar disorder. Thus, although much work ensued regarding potentially helpful descriptions of bipolar symptoms, there is still little consensus.

### 5.2. Duration of Manic Symptoms

Although there are different symptom rubrics that may be used to identify bipolar symptoms in children, researchers also noted that the duration of bipolar symptoms should be considered carefully. Although children may meet the *DSM *symptom criteria for bipolar disorder or be described in a meaningful fashion using one of the rubrics noted above, one of the leading reasons that children may not be diagnosed with bipolar disorder is because they do not meet the stringent *DSM* duration criteria for hypomania and mania [29, 37, 42, 49, 50]. This issue with the duration of criteria can occur with adults as well (e.g., [51]). Nonetheless, children often seem to exhibit a very rapid fluctuation in mood symptoms, especially when such symptoms are comorbid with other disorders [42].

To better classify these symptoms based on duration, Suppes and colleagues [52] modified Kramlinger and Post's [53] definitions of rapid cycling. In particular, they defined rapid cycling as the experience of more than four mood episodes per year, ultrarapid cycling as the experience of more than four episodes per month, and ultraradian cycling as the experience of more than one mood episode per day for at least four days per week [54]. Generally, the *International Society for Bipolar Disorders (ISBD) *Task Force found support for the current definition of rapid cycling [55]. Recent research also supported the presence of ultrarapid and ultraradian cycling in children [50, 54]; however, the *ISBD *Task Force suggested that a dimensional approach to ultrarapid cycling and to the boundary between ultrarapid cycling and mixed episodes may be warranted [55]. Further, Leibenluft and colleagues [46] suggested that an episode theoretically can last only a few hours but that such episodes often are underresearched given the difficulties of examining a span of hours rather than days or weeks. In the context of such findings, Axelson and colleagues [49] proposed that the duration criteria for manic and hypomanic episodes may not be appropriate for children. Nonetheless, use of common definitions of remission for such episodes (e.g., at least four weeks with no more than two depressive or manic symptoms [56]) may be helpful. Thus, the duration and the frequency of mood states should be examined carefully in children.

### 5.3. Neurobiology of Bipolar Disorder

Although neuroimaging studies have fostered some understanding of the pathophysiology of bipolar disorder in adults (albeit with this understanding being based on mixed results from adult studies), less is known about the pathophysiology of bipolar disorder in children. Based on the research that is being done currently, it seemed that there are several target brain areas that have been gaining support in making a contribution to bipolar symptoms in children and adolescents. Similar to adult studies that have suggested a role of subcortical structures in bipolar disorder (e.g., [57]), research suggested that children and adolescents with familial bipolar disorder have smaller volumes in their amygdala relative to those of control children and adolescents [58–60]. Although some studies suggested that children with bipolar disorder do exhibit differences in the volumes of the hippocampus, caudate, or thalamus relative to healthy controls [59], other studies suggested that smaller volume in the amygdala can be accompanied by smaller volume in the hippocampus in children with bipolar disorder [58]. Chang and colleagues [59] also noted that there is a relationship between past use of lithium and valproate and greater gray matter volume in the amygdala.

Other studies also suggested that children and adolescents with familial bipolar disorder may have underlying abnormalities in the regulation of prefrontal-subcortical circuits. For example, Chang and colleagues [61] found that children and adolescents with familial bipolar disorder have greater activation in their bilateral anterior cingulate cortex, left putamen, left thalamus, left dorsolateral prefrontal cortex, and right inferior frontal gyrus during a visuospatial working memory task, whereas children and adolescents in the control group have greater activation in their cerebellar vermis. Other studies also reported that children and adolescents who experienced at least one manic or hypomanic episode have reduced gray matter volume in the left dorsolateral prefrontal cortex, the left accumbens, and the left amygdala (e.g., [62]) and that adolescents with bipolar disorder have decreased volume in the left superior temporal gyrus [63]. Overall, the role of the bilateral anterior cingulated cortex and the dorsolateral prefrontal cortex are not unexpected, given their suspected role in normal mood regulation (e.g., [64]) and in attention (e.g., [65]). More work is needed, however, to clarify the neurobiology of bipolar disorder in children and adolescents.

### 5.4. Comorbid Diagnoses

In addition to issues with symptom duration, frequency of mood states, and neurobiology, the constellation of comorbid symptoms that occur in conjunction with bipolar disorder may require closer examination in children as well. Differential diagnosis may be difficult given that bipolar disorder and other diagnoses include symptoms that occur commonly in children (e.g., irritability). For example, when children present with abnormal mood, irritability, and hyperactivity, there can be substantial discrepancies across health service providers about which diagnosis may be most appropriate [46]. Discussions of differential diagnosis and comorbidity may be facilitated by returning to the four subtypes of childhood-onset bipolar disorder described by Leibenluft and colleagues [46]. As already noted, these subtypes were the narrow subtype, two intermediate subtypes (also depicted as mania-not otherwise specified (short episodes, hallmark symptoms) and irritable mania (full-duration episodes, no hallmark symptoms)), and the broad subtype.

As already noted (via [46]), the narrow subtype can be used to describe children who meet full *DSM-IV-TR* diagnostic criteria for mania, including the duration criteria and characteristic symptoms of mania (e.g., an elevated/expansive mood, an increase in goal-directed activity, and grandiosity [29]). With regard to the intermediate subtypes, the mania-not otherwise specified (short episodes, hallmark symptoms) phenotype characterizes children who experience mania episodes that are shorter than the prescribed duration but who meet the rest of the *DSM-IV-TR* diagnostic criteria for manic episodes. In contrast, the irritable mania (full duration episodes, no hallmark symptoms) phenotype can be used to describe children who exhibit irritability only, rather than elevated/expansive mood, and who meet the rest of the *DSM-IV-TR *diagnostic criteria for manic episodes. Finally, the broad subtype can be used to describe children who have a chronic, nonepisodic illness that does not meet criteria for manic episodes but who still exhibit symptoms of severe irritability and hyperarousal. According to Leibenluft and colleagues [46], these children may never develop a classic mania presentation; however, they still may follow the clinical course of bipolar disorder. Recent research [66] suggested that this phenotype may be distinct from bipolar disorder (e.g., even having different predictors). These descriptions may better capture the symptoms exhibited by children in the context of bipolar disorder.

With these phenotypes in mind, it should be clear that children who have bipolar disorder can experience symptoms that overlap greatly with other childhood disorders [67, 68]. Specifically, research proposed that many children with bipolar disorder also meet criteria for ADHD, major depression, anxiety disorders, ODD, other behavior disorders, and psychosis, even after overlapping symptoms (e.g., distractibility, motoric hyperactivity, and talkativeness) are removed [33, 43, 69–74]. Thus, those working with children should be aware of cooccurring diagnoses, particularly since some distinctions may be more difficult than others.

For example, classifying cooccurring hyperactivity and overaggressiveness may make the bipolar disorder diagnostic picture more difficult, as research suggested that the most common symptoms of bipolar disorder are “excessive energy, decreased need for sleep, impairing elation, and hypersexuality” as well as aggression and irritability ([74, p. 393]; also [46, 75]). Thus, many researchers have been concerned with the overlap between bipolar disorder and ADHD [76, 77] and between bipolar disorder and ODD. In particular, Leibenluft and colleagues [46] suggested that many symptoms in bipolar disorder (e.g., talking excessively, impulsivity, having poor judgment, irritability, often running about or climbing excessively, and distractibility) lack specificity to bipolar disorder and also are present in ADHD [40, 43, 73, 75]. Further, for those researchers who suggested that mania can be chronic (a minority viewpoint), it may be difficult to differentiate bipolar disorder and ADHD if health service providers are looking for specific episodes or mood swings [43].

Nonetheless, Wozniak and colleagues' [43] study provided wider recognition to the similarity between ADHD and childhood-onset bipolar disorder. They claimed that the children with ADHD in their study who exhibited anger, oppositionality, and hyperactive behavior also met *DSM* criteria for childhood-onset bipolar disorder. They recommended that more research be conducted to determine if these children had ADHD, bipolar disorder, or both. In addition, Luby and colleagues [74] found that it was challenging for clinicians to differentiate oppositional behaviors (often found in ODD) from the grandiosity and euphoric mood that is experienced with bipolar disorder (e.g., [75]). As a result, they proposed that health service providers should determine whether the oppositional behaviors are generalized or relationship specific to assist in making a distinction.

Beyond ADHD and ODD, Luby and Belden [35] also suggested that preschoolers with bipolar disorder experienced a greater severity of depression and more comorbidity than those preschoolers with major depressive disorder. Nonetheless, many of these symptoms also were present in other psychiatric disorders, making differential diagnosis difficult [40, 73]. More recently, in order to better distinguish affective and behavioral disorders first seen in childhood, APA proposed a new diagnostic category, disruptive mood dysregulation disorder (DMDD, previously known as severe mood dysregulation and temper dysregulation disorder, e.g., [66]), for *DSM-5* [30]. DMDD is described as a disorder that is characterized by extreme, recurrent temper outbursts (both physical and verbal) that are disproportionate to the provocation or stressor prompting the outburst and inconsistent with children's developmental level [30]. DMDD is proving to be a controversial addition to the *DSM-5*, with some researchers voicing significant reservations about this disorder (e.g., [78]). Given its reference to dysregulation of mood and emotion, however, this disorder may aid in the facilitation of research on the causes, features, and treatment of childhood-onset bipolar disorder as researchers continue to try to differentiate one disorder from the other given that bipolar disorder is proving to be a disorder that is distinct from DMDD (e.g., [66]).

Nonetheless, childhood-onset bipolar disorder now is recognized and does share features with adult-onset bipolar disorder. For example, researchers suggested that there is continuity in symptoms across adolescence [41] and into adulthood [79]. Further support for the continuity of symptoms over the course of development was supported by family studies comparing children with bipolar disorder to their parents who had bipolar disorder via neuroimaging [61] and with genetic studies [80]. Findings in these studies suggested that bipolar disorder with an onset in childhood may be genetically loaded and homogenous [80], allowing for the documentation of similarities across family members.

Given the challenges that are present when identifying and diagnosing bipolar disorder in children (particularly preschool children), it may prove beneficial to consider Robins and Guze's [81] research validation paradigm for identifying a valid syndrome (e.g., [37, 46, 82, 83]). They proposed that a syndrome should have a reliable clinical description, consistent support from research studies, differentiation from other clinical disorders, evidence from follow-up research indicating that clinical outcomes of patients are similar or do not meet criteria for another disorder, and proof of increased prevalence rates in family studies. Although specific considerations that are necessary for the diagnosis of bipolar disorder in children are not completely clear (beyond actual *DSM* criteria), the fact that these children experience significant impairment in family, school, and social functioning should be emphasized. In addition, these children exhibit poor global functioning, utilize mental health care facilities, and attempt suicide more frequently [34], suggesting the severity of their symptom presentation.

With this information in mind, more specificity and sensitivity could be achieved for bipolar disorder diagnoses in children. For example, a meta-analysis conducted by Kowatch and colleagues [73] suggested that the most frequently occurring symptoms of mania in children include increased energy, distractibility, and pressured speech, with 89%, 84%, and 82% of children displaying these symptoms, respectively. Additionally, 78% of children experienced grandiosity, 72% of children experienced decreased need for sleep, 70% of children experienced elation/euphoria, and 69% of children exemplified poor judgment. In contrast, the rarest symptoms experienced by children with bipolar disorder were flight of ideas (56%) and hypersexuality (38%, [73]). Other research suggested that about one-fifth of children with bipolar disorder experience delusions or hallucinations during a mood episode. Further, psychosis was more common among children meeting criteria for bipolar disorder than among children with ADHD [40, 69, 84]. Based on these findings, particular specific symptoms also may prove to be distinguishing factors when making a bipolar disorder diagnosis in children.

### 5.5. Irritability

One symptom that may deserve more consideration than others when attempting to understand bipolar symptoms in children is irritability, emphasizing an important component of the *DSM-IV-TR* bipolar criterion of “a distinct period of abnormally elevated, expansive, or irritable mood” [29, 68]. In fact, the rationale for the intermediate subtypes described above was that irritability is not pathognomonic to bipolar disorder but rather is ubiquitous in childhood psychopathology ([46, 66, 69, 75] and also can occur in typically developing children). For example, irritability may be present in at least six childhood disorders (e.g., major depressive disorder, generalized anxiety disorder, posttraumatic stress disorder, ADHD, ODD, conduct disorder [68, 85]). As a result, it will be important to determine whether children's irritability is part of bipolar disorder or some other disorders [85, 86].

Clearly, some research suggested that irritability is an important marker in childhood-onset bipolar disorder as it may be a sign of severity (e.g., [87]). For example, Biederman and colleagues [69] suggested that irritability in children with bipolar disorder is “very severe, persistent, and highly disabling, and often associated with violence” (p. 54). Further, in their meta-analysis, Kowatch and colleagues [73] suggested that 81% of children with bipolar disorder experienced irritability. Nonetheless, given that no *DSM* diagnosis captures the symptomatology of predominantly severe, nonepisodic irritability well, more attention is needed in classifying this symptom [68]. In particular, for symptoms of irritability to be useful in making a diagnosis, definitions of irritability that account for the context in which it occurs and its episodicity and chronicity would be required. Currently, the *DSM* does not define irritability, making it difficult for health service providers to clearly identify this symptom in children [29, 30, 66, 68, 88]. Other definitions do exist in the literature, however. For example, the *K-SADS* [89] defined irritability as “anger, crankiness, bad or short tempered, resentment or annoyance, touchy or easily annoyed” [88, p. 410]. Further, the *Diagnostic Interview Schedule for Children* (*DISC* [90]) defined irritability as “cranky, angry toward people you had no reason to, talking back, or temper tantrums” [62, p. 410]. The *Children's Depression Rating Scale* [91] also defined irritability as “grumpy, crabby, talked back, sassy, wouldn't do something your parents asked you to do” [62, p. 410]. Nonetheless, it is difficult to identify this symptom with any degree of certainty [62].

Others emphasized the importance of irritability in establishing bipolar disorder diagnoses in a manner more consistent with the suggestions of Wozniak and colleagues [43]. In such cases, a diagnosis would be recommended if children meet *DSM* criteria with irritability as a core symptom and without the presence of elation, grandiosity, and episodicity. Nonetheless, some research suggested that there may not be many differences between those children who exhibit episodic irritability versus those who exhibit episodic elation. In particular, a study by Hunt and colleagues [92] noted that these two groups of children do not differ in their rate of psychiatric comorbidities, severity or duration of illness, or family history of mania. The main difference between these two groups was that children who exhibit irritability have more symptoms of depression and alcohol abuse and that children who exhibit elation have more manic symptoms. Still others used some combination of these recommendations by considering irritability to be a core symptom only if it is accompanied by elated mood or grandiosity [46]. Given these inconsistencies, research is needed to provide further clarity regarding the relative importance of irritability, grandiosity, and elation in making a diagnosis of bipolar disorder in children [93].

### 5.6. Emotion Regulation

Although not yet examined widely in the context of bipolar disorder in children, emotion (dys)regulation also may prove helpful in delineating the bipolar symptoms that children are experiencing (e.g., [94]; see [95], for a review; see [96], for further information). Those who are diagnosed with bipolar disorder obviously experience difficulties in their emotion regulation [33, 97, 98]. This is true especially for preschool children, a population that recently received much attention and debate concerning the diagnosis of bipolar disorder [33, 46, 74, 88, 98–101]. For example, despite the key differences between the subtypes of childhood-onset bipolar disorder proposed by Leibenluft and colleagues [46], the children in each of these subtypes still experience a shared impairment in emotion regulation [33, 99]. As a result, emotional dysregulation may be viewed as an important characteristic of bipolar disorder [46], while also considering the changes in energy [30] and cognitive disruptions [102] that can accompany a bipolar disorder presentation.

Although Leibenluft and colleagues [46] conceptualized childhood-onset bipolar disorder as having phenotypes, these phenotypes also can be viewed as being on a spectrum (similar to the spectrum that now is used for pervasive developmental disorders; also see [103]). Located at one end of the bipolar spectrum is the narrow subtype, as it is characterized by the classic manic and depressive symptoms usually manifested in adults. On the other extreme of the bipolar spectrum lies the broad subtype, characterized by highly impairing, chronic, and nonepisodic irritability [66]. Such a bipolar spectrum would suggest that bipolar disorder consists of varying degrees, severities, and presentations of emotional dysregulation and other symptoms. Generally, it is advantageous for health service providers to use a bipolar spectrum with children because a group of specific categorical bipolar diagnoses may not encompass all of the children needing treatment for bipolar symptoms, especially if they do not fit exact criteria. Thus, although emotion regulation is not addressed or included explicitly in the *DSM-5*, it may prove beneficial to examine emotion regulation when considering a diagnosis of bipolar disorder in children [29, 66]. Nonetheless, Luby and Belden [33] indicated that emotion regulation is difficult to operationalize and quantify.

Thus, in order to discuss emotion regulation in the context of bipolar disorder in children, a definition would be needed. In its most basic sense, emotion regulation involves individuals' ability to control their reactions, affect, emotions, and mood [33, 97, 99]. Further, Dickstein and Leibenluft [99] explained in greater detail that emotion regulation consists of three major processes, including being able to detect a stimulus in the environment, to generate an emotional response, and to process or control emotion. When stated simply, it becomes evident that children who have a disorder on the bipolar spectrum experience emotional dysregulation, as evidenced by their increased emotional lability, intensity, and reactivity [74]. Other researchers discussed emotion regulation from a neurobiological perspective, however. For example, the prefrontal regions of the brain play an inhibitory role in emotion regulation, whereas subcortical regions (that encode and represent information about emotions) experience decreased activation (e.g., [97]). Alloy and Abramson [104] also suggested that a behavioral approach system (BAS) dysregulation theory may further the understanding of the psychosocial and biological features of bipolar disorder, with the lability in mood, energy, motivation, cognition, and activity that is experienced being associated with high BAS sensitivity (in contrast to the effects of an individual being overregulated). Researchers noted, however, that much controversy still surrounds the topic of emotion regulation, as there has not been unanimous agreement on its exact definition or its proper measurement and assessment [33, 99].

To further complicate matters, emotional dysregulation is part of typical childhood development and is present in all children at certain points in time. Nonetheless, emotional dysregulation becomes indicative of a disorder when it clearly is impairing to children's functioning, is developmentally inappropriate for children's age, and is inappropriate or out of proportion relative to the situational or environmental triggers that children are experiencing [74, 88]. Once emotional dysregulation is considered in terms of this developmental context, it may become a useful consideration in the context of bipolar disorder in children. In fact, Luby and Belden [33] suggested that there are actually two forms of emotional dysregulation that may be important for childhood-onset bipolar disorder. The first form of dysregulation can be described as *underregulation *and is evident when children are unable to regulate their emotions enough. In this form of dysregulation, emotions become out of control. Emotional underregulation also is related to *behavioral disinhibition*, where children experience difficulties in controlling and modifying their inappropriate behaviors and experience impulsivity, absence of restraint, high approach, and disinhibited behaviors and speech in novel situations. Emotional underregulation and behavioral disinhibition may lead to externalizing problems in children, even in young children [33, 100, 101]. Conversely, the second form of emotional dysregulation can be described as *overregulation* and is evident when children regulate their emotions too much. As a result, their emotions are dulled or dampened [33]. Luby and Belden [33] suggested that emotional overregulation also is related to *behavioral inhibition* and may lead to internalizing problems in children, even in young children [33, 100]. Overall, Hirshfeld-Becker and colleagues [100] suggested that examining the overlap and relationship between emotion regulation and behavioral inhibition and disinhibition should prove to be a productive area of research in the future.

Certainly, discussing the effects of both emotional underregulation and overregulation brings to mind the hallmark features of bipolar disorder in children. Bipolar disorder is characterized by both highs (mania) and lows (depression), often exhibited by children as externalizing and internalizing behaviors, respectively [29, 33, 74]. Luby and Belden [33] conceptualized childhood-onset bipolar disorder as the combination of emotional underregulation and overregulation contributing to such problematic behaviors. They suggested that externalizing problems in children (particularly young children) result from the underregulation of negatively valenced emotions (e.g., anger) and affect. Additionally, they proposed that internalizing problems in children (particularly young children) may be caused by a constellation of emotional regulatory and behavioral difficulties, including children's overregulation of positively valenced emotions (e.g., joy), underregulation of negatively valenced emotions (e.g., sadness), and inhibited behavioral ability to generate feelings of pleasure [33]. The simultaneous presentation of such externalizing and internalizing behaviors can be indicative of rapid cycling and mixed episodes, both of which are thought to be more common in children [93, 99]. Emotional dysregulation also may be used to explain more classic manifestations of bipolar disorder in children, however. For example, children may underregulate positively valenced emotions (e.g., happiness), resulting in elation or the elevated/expansive mood characteristic of mania in adults [33].

Although research is beginning to consider emotional dysregulation as a symptom of childhood-onset bipolar disorder, emotional dysregulation also may be a prominent feature of many other childhood disorders (e.g., ADHD, ODD, CD). Therefore, health service providers still must use caution when examining children whom they suspect may lie on the bipolar spectrum due to difficulties with emotion regulation. The symptom of emotional dysregulation alone is, of course, not enough to diagnose children with bipolar disorder (or any other disorder, for that matter). Several researchers suggested, however, that emotional dysregulation and behavioral disinhibition are likely early markers for bipolar disorder [98, 101]. Although a diagnosis cannot be made based only upon the presence of an early marker or prodrome, such evidence may allow health service providers to observe closely those children who may be most likely to be at risk due to emotional dysregulation and to monitor them for the later bipolar symptoms. Such monitoring also will allow researchers to conduct longitudinal studies in the area of emotional dysregulation and bipolar symptoms.

To provide a further narrowing of focus, emotional reactivity may be a particularly important component of emotional dysregulation. In fact, children with bipolar disorder appear to experience difficulties with increased emotional reactivity. For example, Chang and colleagues [105] suggested that children who are at risk for developing bipolar disorder experience difficulties in reactivity. They found that children of parents who have bipolar disorder showed difficulties with emotional reactivity by continuing to exhibit anger or depression despite being consoled by another individual [105]. Further, based on parent reports, Luby and colleagues [74] discovered increased emotional reactivity in children with bipolar disorder as well. For example, they found that, after a joy-inducing stimulus event, preschoolers who were diagnosed with bipolar disorder expressed significantly higher levels of joy than typically developing peers at 30 and 60 minutes after the event. These preschoolers retained these significantly higher levels of joy at lunchtime and at dinnertime. Preschoolers with bipolar disorder also expressed significantly higher levels of sadness after a sadness-inducing event at 30 minutes after the event. Further, these preschoolers expressed significantly higher levels of anger initially and 30 minutes after an anger-inducing event [74]. Such findings were consistent with those of Gruber et al. [106], who found that interepisode bipolar disorder in adults was related to greater rumination about positive and negative emotions and that this rumination was related to illness course.

Luby and colleagues' [74] study also elicited some intriguing results that may help researchers and clinicians differentiate between preschoolers with bipolar disorder and preschoolers with other disruptive behavior disorders. Preschoolers with bipolar disorder expressed significantly higher levels of joy than disruptive preschoolers at 30 and 60 minutes after a joy-inducing event and at lunchtime. Preschoolers with bipolar disorder also expressed significantly higher levels of sadness 30 minutes and 60 minutes after an event inducing that feeling and at bedtime. Additionally, preschoolers with bipolar disorder expressed significantly higher levels of initial anger and anger at 30 minutes and 60 minutes after an event inducing this emotion [74]. This evidence demonstrated that young children with bipolar disorder experience difficulty with emotion regulation and intensity over long periods of time. These differences in reactions pointed to the increased emotional reactivity and intensity displayed by young children with bipolar disorder but not with other disruptive behaviors. Thus, observations of emotional reactivity may aid in complex differential diagnosis [74] although it also should be recognized that emotional regulation is not specific to bipolar disorder and may prove to be important to other diagnoses as well (e.g., [107]).

## 6. Valuable Perspectives on Assessment

Given the nature of the symptoms that are displayed by children struggling with bipolar symptoms, the commonality of certain symptoms across different disorders (e.g., bipolar disorder versus ADHD), and the need for more attention regarding other characteristics that potentially may be helpful in making a bipolar disorder diagnosis (e.g., irritability, emotional dysregulation), the assessment of bipolar disorder in children likely will be challenging. The purpose of this section is to highlight basic guidelines for assessing bipolar disorder in children. As a means of assisting with the assessment of bipolar symptoms, AACAP [108] published a Practice Parameter in 2007 for assessing bipolar disorder in children. Specifically, four recommendations were provided for the assessment of bipolar disorder in children. The first recommendation suggested that *DSM* criteria should be followed when diagnosing mania or hypomania [108]. Because symptoms such as irritability and emotional lability are seen across many disorders of childhood and also can be seen in typically developing children, adhering closely to the operational definitions of mania and hypomania provided in the *DSM* may foster a clearer diagnostic picture for children. In particular, close attention to the pattern and duration of bipolar symptoms and how these symptoms relate to sleep, motor activity, and changes in cognitive functioning can provide key information that health service providers can use to parse out the likelihood that such symptoms may be due to bipolar disorder or some other disorders [108].

The second recommendation in the Practice Parameter [108] was that bipolar disorder not otherwise specified should be used for children who display an atypical duration of bipolar symptoms. Given that the course of symptoms in children can vary greatly relative to *DSM *criteria and that interventions for bipolar disorder in children resemble those provided to adults (although there is still uncertainty regarding the effectiveness and safety of using such an adult-oriented approach), this recommendation may facilitate children receiving interventions that may prove useful in alleviating the bipolar symptoms that they are experiencing. The Practice Parameter further reported that children who are diagnosed with bipolar disorder not otherwise specified have high rates of comorbid disorders, both in the internalizing and externalizing domains, and that the moods of these children are generally volatile and reactive [108]. As a result, this recommendation implies that health service providers must understand clearly other factors (e.g., situational triggers, reinforcement of outbursts, communication difficulties, and risk factors) that can facilitate the differential diagnosis of bipolar disorder versus other disorders (e.g., ADHD) and assist in identifying other comorbid diagnoses as well.

The third recommendation provided by the Practice Parameter [108] suggested that problems associated with bipolar disorder need to be assessed closely. These problems included suicidality, commonly comorbid disorders, psychosocial stressors, and medical problems. As a result, a comprehensive assessment that includes a thorough understanding of children's developmental abilities, cognitive functioning, and speech and language abilities (all of which may be particularly important for children, particularly preschool children) is warranted when assessing bipolar disorder. Such information also will facilitate a comprehensive treatment plan.

The final recommendation provided by the Practice Parameter [108] was that the diagnosis of bipolar disorder should be used cautiously in young children. The Practice Parameter underscored that the “diagnostic validity of bipolar disorder in young children has yet to be established” [108, p. 115]. Children who present with mood and behavior problems may be experiencing various environmental and biological factors that may account for such problems (e.g., developmental delays, psychosocial stressors, parent-child relationship problems, and children's own temperament [108]). Given that pharmacological interventions are the standard for bipolar disorder and given the lack of research on the safety and efficacy of such interventions for children, this diagnosis and consequent interventions should be considered carefully.

In addition to the recommendations offered as part of the Practice Parameter [108], Baroni et al. [109] also provided detailed guidelines regarding the assessment of mania in children. Their recommendations suggested that both parents and children should be interviewed together and separately (when their age is appropriate to do so), that screening for mania should include questions about distinct periods of mood change and associated symptoms, and that the period of time in which children display euphoria or heightened irritability should be identified. They also suggested that a developmental perspective should be used to evaluate the severity of the symptoms and whether they are clinically relevant, that it should be determined whether elation is comparable to or greater than positive affect during experiences that could cause great excitement (e.g., Christmas morning), that it should be determined whether manic symptoms occur during the mood change, and that grandiosity versus oppositionality should be considered by thinking about whether the behavior is a change from the norm and if it occurs along with other new symptoms. Lastly, they suggested that a distinction should be made between a decreased need for sleep and insomnia. Thus, many considerations for assessing bipolar disorder in children generally were offered.

Nonetheless, other considerations may be needed as well. Of particular importance for children, particularly preschool children, assessment should be an ongoing process in which children's symptoms are monitored over time [110]. In fact, children's behavioral history should be collected so that a baseline for behavior can be established (e.g., [110]). This baseline will be important for understanding whether children's mood changes are significant. An important visual tool to consider in this process is a mood log, in which retrospective and prospective data about children's behaviors and symptoms can be tracked [111]. It also was recommended that, at the time of scheduling an assessment for a suspected bipolar disorder diagnosis, parents should be asked to log their children's symptoms for at least two weeks [112]. In this way, patterns of symptoms can be documented. Finally, practitioners should utilize multiple informants when possible [111, 113], as multiple informants likely will provide useful information. Nonetheless, it will be particularly important to consider parents' reports, as this information may provide the best diagnostic accuracy [113].

In addition, a detailed historical interview should be conducted, including symptom characteristics over time, responses to intervention, and familial psychiatric history. Such information can help health service providers further understand children's symptoms and whether these symptoms are truly indicative of bipolar disorder. Family psychiatric history, in particular, is a well-established risk factor for bipolar disorder, with children of parents who have bipolar disorder being at five times higher risk for developing the disorder than other children [114, 115]. Other important information for assessing bipolar disorder in children may include mood lability (e.g., rapid changes in episodic mood from manic to depressive symptoms versus the cooccurrence of depressive and manic symptoms), psychotic features that are mood contextual (e.g., delusions or hallucinations), and episodic aggressive behavior that is reactive in nature [116]. Such recommendations will better focus assessments of bipolar disorder in children.

Regarding specific assessment methods, other helpful papers exist detailing evidence-based methods (e.g., [39, 117]) and specific assessment tools along with their psychometric properties (such as the *Kiddie Schedule of Affective Disorders and Schizophrenia (KSADS)*, the *Diagnostic Interview Schedule for Children (DISC)*, and the *Child Mania Rating Scales*; e.g., [111, 116, 117]). Some brief examples will be overviewed here. Generally, structured or semistructured interviews are used to generate diagnoses for research (and sometimes for clinical) purposes. As listed above, the KSADS often is used for this purpose with parents of children and adolescents and sometimes with adolescents themselves. Although the KSADS has sufficient coverage of bipolar symptoms [118], administration of structured interviews can take a significant amount of time, resulting in a significant expense and a lack of feasibility for assessment situations [119].

As a result of these limitations of structured interviews, a variety of rating scales may be used instead. At times, more broad-based rating forms, such as the Child Behavior Checklist (CBCL, Achenbach and Rescorla [120]), may be used, particularly as some suggested that the CBCL can be of assistance in identifying bipolar symptoms in children via the use of a constellation of its subscales (i.e., the attention problems, aggressive behavior, and anxious-depressed subscales [121]). A potentially more useful and expedient option, however, may be to use scales that are created specifically to detect bipolar symptoms in children via ratings provided by parents (see [119]). There are several examples of such rating scales that have good psychometric properties, including the Parent General Behavior Inventory (GBI [113]), the Parent Young Mania Rating Scale (YMRS [122]), and the Child Bipolar Questionnaire (CBQ [119]). Some studies suggested that specific parent rating scales, such as the GBI and the YMRS, produce fewer false alarms relative to broad-based scales, such as the CBCL [110]. Nonetheless, the GBI and the YMRS only assess manic and hypomanic symptoms, whereas the CBQ was developed to assess manic, depressive, and comorbid symptoms [119]. For the GBI and the YMRS, there are also self-report versions of these scales for children and adolescents; however, such scales may have more limited utility for younger children. Overall, the assessment of bipolar disorder should be both comprehensive and longitudinal in nature [111].

## 7. Insights into Potential Therapeutic Interventions for Children

Once a thorough assessment has been completed, options for therapeutic intervention should be explored carefully. Although the literature on appropriate interventions for children with bipolar disorder is growing, more research is needed to further the understanding of which interventions will work best for which children. As a result, some of the options that are available for older children and adolescents are examined here in an effort to provide potential insight into those options that may prove efficacious for children, particularly preschool children, and to draw attention to the fact that more research is needed.

### 7.1. Medications

Although psychopharmacological interventions may be considered most efficacious generally in the treatment of bipolar disorder, much of the available information regarding the efficacy of psychopharmacological interventions with childhood-onset bipolar disorder was extrapolated from the adult literature. As such, there were few studies that directly addressed the effects of these medications in children and adolescents [38]. There were even fewer studies that address the efficacy of these medications in preschool children with bipolar disorder. Generally, however, a theme in prescription recommendations was *primum non nocere *(i.e., first do no harm [123]). Nonetheless, it will be important to further investigate the long-term effects, efficacy, and side effects of these medications in children, particularly in preschool children.

With regard to the research available for children and adolescents generally, the most commonly prescribed medications for children and adolescents who exhibit bipolar symptoms were lithium, antiepileptic medications (i.e., divalproex sodium and carbamazepine), and atypical antipsychotic medications (i.e., risperidone, olanzapine, quetiapine, ziprasidone, and aripiprazole). Of these medications, lithium is approved by the FDA to treat mania in children who are 12 years of age and older, whereas risperidone and aripiprazole are approved for children who are 10 years of age and older [124]. Much of the research on children and adolescents indicated that, when used alone, these medications are relatively effective (see [124, 125]), generally safe, and well tolerated [126]. Nonetheless, some suggested that atypical antipsychotics may be more effective than mood stabilizers (although they may promote more adverse events) when used in children and adolescents (e.g., [127–129]). For example, Geller and colleagues [128] reported that 279 children and adolescents who ranged in age from 6 to 15 years and who were diagnosed with bipolar I disorder had higher response rates to risperidone than to lithium and to divalproex sodium during the course of a controlled, randomized, no-patient choice, and eight-week protocol. Nonetheless, risperidone may promote serious metabolic effects [128].

Beyond using one medication in an effort to treat bipolar symptoms, medications showed promising results when they were utilized in combination, whether that combination included multiple mood stabilizers [130] or mood stabilizers augmented with atypical antipsychotic medication [126]. For example, Findling and colleagues [131] indicated that children and adolescents who ranged in age from 5 to 17 years and who met criteria for bipolar I disorder or bipolar II disorder were treated safely and effectively using a combination of lithium and divalproex sodium. Once stabilized on this combination of medications, some children and adolescents could continue to be maintained and do well on either lithium or divalproex sodium; however, many children and adolescents experienced relapse or adverse events [132]. In order to effectively combine these medications, however, multiple algorithms were proposed that systematically introduced both mood stabilizers and antipsychotics according to the individual needs of the children and adolescents and their respective treatment responses [112, 133]. Overall, preliminary results using this algorithm approach demonstrated success in reducing symptoms of childhood-onset bipolar disorder [133], but, again, more research is needed on such approaches.

In the context of *primum non nocere*, it should be noted that, although these medications are used widely and are beneficial in treating bipolar symptoms, they often are accompanied by adverse side effects, including gastrointestinal distress (e.g., nausea, vomiting), headache, cognitive dulling, hypothyroidism, tremors, and extrapyramidal side effects [112, 124, 134]. Further, one particularly worrisome and common side effect is weight gain. Others also documented metabolic effects [135] and cardiovascular risks (although it is unclear if these risks may be present prior to the administration of medications [136]). Given such side effects, it was recommended that children and adolescents be monitored closely, particularly as these side effects can lead to a number of long-term effects [112, 137]. Further, such significant side effects also suggested that examination of alternative treatment options, such as flax seed oil and omega-3 fatty acids, is warranted [138].

Often children and adolescents with bipolar symptoms also exhibited symptoms that were consistent with other comorbid conditions, such as ADHD, disruptive behavior disorders, and anxiety disorders [86]. Treatment of these disorders raises additional concerns, particularly when a comorbid condition also requires intervention with psychotropic medications. Most often, clinicians recommended that bipolar symptoms be stabilized before any additional medications are introduced to address comorbid conditions [112, 125]. Additional medications that were added once bipolar symptoms were stabilized, particularly psychostimulant medications used to treat ADHD, showed promising results [130, 137]. In particular, mixed amphetamine salts were significantly more effective than placebo for treating ADHD symptoms in 6 to 17 year olds with a bipolar I diagnosis [139]. Nonetheless, some medications (i.e., antidepressants) should be monitored closely. Although many suggested that antidepressant-induced bipolar symptoms may occur uncommonly (e.g., [140, 141]), some children and adolescents may be susceptible to such symptoms [142].

As previously mentioned, although the use of psychotropic medications is increasing in the preschool population, these medications have not been studied adequately [143, 144]. In the limited research that was conducted with preschool children, several of the commonly used medications that were effective in older children also appeared to be effective in this population. Specifically, studies investigating risperidone, olanzapine, valproate, divalproex sodium, and carbamazepine all showed positive results [70, 145–147]. In contrast, lithium may not be as effective as these other medications [126]. Further studies suggested that psychostimulant medications used to treat comorbid ADHD symptoms prompt clinically significant changes in preschoolers' behavior and are well tolerated by individuals in this age group [148]. To verify such results, more research using randomized control trials is warranted.

### 7.2. Cognitive-Behavioral and Psychosocial Interventions

Despite the recommendation that psychotherapeutic interventions be used as an adjunct to psychopharmacological interventions in order to address psychosocial factors that may accompany bipolar symptoms [137], research investigating the use of psychotherapeutic interventions with childhood-onset bipolar disorder was sparse. Overall, the primary psychotherapeutic interventions for childhood-onset bipolar disorder focused on the role of high emotion expression in families (e.g., critical remarks, hostility, and overprotection). Specifically, these treatments posited that these behaviors had detrimental effects on children with bipolar disorder and may encourage negative reactions in children (e.g., temper tantrums, self-destructive behavior). These negative reactions, in turn, exacerbated further high emotion expression behaviors [149, 150]. Thus, many of the psychotherapeutic interventions available sought to address dysfunctional communication styles within families that might exacerbate the presence of bipolar symptoms and to teach families about the management of bipolar disorder via psychoeducation [151] in either individual or group settings [149]. Additional areas of focus for psychotherapeutic intervention included relapse prevention, individual psychotherapy, social functioning, and academic functioning [137].

Generally, research suggested that cognitive-behavioral interventions (i.e., those that address the aforementioned topics) are feasible and efficacious with children and adolescents with bipolar disorder [152, 153]. In fact, researchers are beginning to develop specific psychotherapeutic programs that utilize the cognitive-behavioral theoretical framework. One such example is the “Think Effectively about Mood Swings (TEAMS)” program [154]. This program is based on an integrative cognitive model that suggests that multiple and extreme appraisals of internal state changes and the impact that these changes have on behavior, physiology, and the environment actually maintain and exacerbate bipolar symptoms. By addressing the extreme positive and negative appraisals of internal state changes through cognitive-behavioral therapy, bipolar symptoms were improved [154]. Although this particular program was examined in a small sample of adults, such programs may have applicability for children (and preschool children) if adapted for their developmental levels.

An additional therapy that may prove useful for treating symptoms of bipolar disorder in children may be an adaptation of Dialectical Behavior Therapy (DBT), a treatment designed originally for Borderline Personality Disorder [155], given its focus on mindfulness and distress tolerance skills. For example, Goldstein and colleagues [156] conducted a one-year open trial of DBT with adolescents who had bipolar disorder using both family skills training and individual therapy. Although their open trial included only ten adolescents, their study suggested that the feasibility and acceptability of using DBT were high and that the adolescents exhibited significant improvements in their symptomatology (e.g., suicidality, self-injurious behavior, emotional dysregulation, and depressive symptoms) in conjunction with the intervention [156].

Finally, new therapies are beginning to focus on the difficulties that children and adolescents with bipolar disorder have with the rhythmicity of their behaviors (e.g., in sleep, behaviors, and mood episodes). One such therapy is Interpersonal Social Rhythm Therapy (IPSRT [157, 158]), a therapy that was derived from interpersonal psychotherapy for depression and behavioral intervention for social rhythm and sleep-wake regulation. The underlying premise of this therapy is that individuals with bipolar disorder need to recognize the relationship between disruptions in social rhythms and the onset of previous mood episodes. They then can use behavioral interventions to make improvements in their social rhythms and sleep-wake regularity [158]. Research suggested, thus far, that IPSRT is a useful adjunct to pharmacological interventions, that it can extend the time between mood episodes, and that it can improve individuals' social rhythms [158].

### 7.3. Family Interventions

In addition to addressing these treatment areas, family interventions also can be helpful, particularly for families who are raising children with bipolar disorder. For example, Multifamily Psychoeducation Groups (MFPG) and Individual Family Psychoeducation (IFP) were provided as a potentially useful treatment for childhood-onset bipolar disorder. MFPG and IFP included education about bipolar symptoms and symptom management, the improvement of communication skills among family members, and the increase of support among family members [151]. MFPG was designed for implementation in group settings. Such settings provide the opportunity for children to augment their social skills and for parents to gain support from other families. Each session began with all of the families together before children and parents participated in their own groups. The child and parent groups then covered similar topics but addressed the specific needs of each group [149]. For parents, topics included education regarding their children's bipolar symptoms, medications, community services, family communication, problem solving, coping skills, and symptom management. For children, similar information was provided but focused more heavily on improving children's skills through applied practice [159].

IFP was developed so that the needs of families could be addressed on an individual basis. This treatment protocol utilized alternating child and parent sessions (rather than completing both sessions concurrently). The content of treatment sessions was similar to that provided in MFPG. In contrast, families also were provided with additional information regarding healthy habits (e.g., sleep hygiene, nutrition, and exercise). In addition, although this treatment was manualized, several sessions were built into the treatment protocol so that topics could be personalized for individual families [159]. Both the group and individual formats of this treatment effectively demonstrated symptom decreases in children who ranged in age from 8 to 11 years [151, 160]. Given these findings, this treatment protocol should be examined further for families who have children with bipolar disorder.

An additional family treatment, the Family Focused Treatment (FFT) protocol, was adapted for use in adolescents from a treatment that already had proven successful in adult patients. The goals for this treatment protocol focused on increasing the understanding of mood symptoms, improving medication compliance, enhancing communication skills, and reducing social impairment. Treatment sessions were divided into three modules that included psychoeducation, communication skills, and problem-solving skills. During the psychoeducation module, families were provided with information regarding symptoms, mood tracking, and risk factors. Families also developed a tailored intervention plan to prepare them for instances of symptom relapse. During the communication and problem-solving skills modules, families were provided with strategies to decrease negative communication styles and to increase cooperative family problem solving. Research indicated that this treatment demonstrated significant symptom reduction in adolescents [150]. Thus, with adaptations for families with children, this treatment protocol may have some utility.

As a final family intervention example, the Child and Family Focused Cognitive-Behavioral Therapy (CFF-CBT) protocol was developed as a downward extension of FFT for children (i.e., those who ranged in age from 8 to 12 years) and their families [133]. The treatment protocol incorporated cognitive-behavioral techniques (e.g., problem solving, positive self-view), interpersonal techniques, and psychoeducation. Sessions were structured around children only, parents only, and combined child and parent activities. This treatment protocol also emphasized the inclusion of siblings by providing them with information regarding childhood-onset bipolar disorder in an effort to increase their empathy for their affected siblings. Finally, the therapist worked with the children's school to provide education and appropriate school-based interventions. Efficacy research demonstrated symptom reduction when this treatment protocol was provided both individually [133] and in groups [153]. Additionally, this treatment was somewhat effective with children who were as young as 6 and 7 years of age [153]. These gains were sustained when a maintenance phase was added to the treatment. This maintenance phase included psychotherapeutic booster sessions and assistance with medication management [161]. Such family interventions may hold particular promise for use with families who have children with bipolar disorder given the strong evidence base of these interventions.

### 7.4. Treatment Summary

Although no interventions were validated for use with children, particularly preschool children, who have bipolar disorder, many of the techniques that were used in the aforementioned intervention programs could be useful, particularly if they were modified to be more developmentally appropriate (e.g., accounting for potential cognitive limitations, emphasizing more behavioral components). Specifically, it would be necessary for any proposed treatment to emphasize the involvement of the entire family, particularly their caregivers. Because children rely so heavily on these individuals, it would be imperative for caregivers to understand the nature of bipolar disorder and the necessary actions for effective intervention [162]. Additionally, given the difficulties that are inherent in caring for children with bipolar disorder, it would be likely that caregivers would need a great deal of emotional support. Although children may not be able to participate in all of the cognitive-behavioral aspects of current treatments due to the potential for developmentally limited cognitive abilities, children still could benefit from instruction in emotion regulation strategies, distress tolerance skills, and developmentally appropriate information about their bipolar disorder. Further, because research suggested that children, particularly preschool children, can suffer from significant impairments in communication, daily living, and social skills [33], additional training via behavioral interventions in these areas would likely be very beneficial.

## 8. Conclusions

Although *DSM *criteria for bipolar disorder may have limitations in the context of diagnosing bipolar disorder in children, it is clear that children are receiving this diagnosis. Nonetheless, to improve the developmental appropriateness of the diagnostic criteria that are currently available for bipolar disorder when used in children, particularly preschool children, other considerations should be made. Clearly, the subtypes described by Leibenluft and colleagues [46] and the idea of a bipolar disorder spectrum may be helpful in more carefully defining bipolar disorder in children; however, the prevalence of certain symptoms at young ages and the degree of emotion dysregulation that children, particularly preschool children, may exhibit also should be considered. As adjustments are made in the diagnostic criteria used to label bipolar disorder in children, further recommendations to the assessment process can be added to those already provided by the Practice Parameter [108] and by Baroni and colleagues [109]. Most importantly, however, more progress needs to be made in developing and identifying those interventions, whether via pharmacological interventions and/or family-based cognitive-behavioral interventions, that will treat the symptoms exhibited by children with bipolar disorder most efficaciously. It is only through clearly defining, identifying, and consequently remediating these bipolar symptoms that children can benefit from a more positive trajectory as they proceed through their developmental milestones into later childhood, adolescence, and ultimately adulthood.
